# The Evaluation of AREG, MMP-2, CHI3L1, GFAP, and OPN Serum Combined Value in Astrocytic Glioma Patients’ Diagnosis and Prognosis

**DOI:** 10.3390/brainsci10110872

**Published:** 2020-11-19

**Authors:** Rūta Urbanavičiūtė, Kęstutis Skauminas, Daina Skiriutė

**Affiliations:** Laboratory of Molecular Neurooncology, Neuroscience Institute, Lithuanian University of Health Sciences, Eiveniu str. 4, LT50161 Kaunas, Lithuania; kestutis.skauminas@lsmuni.lt (K.S.); daina.skiriute@lsmuni.lt (D.S.)

**Keywords:** cancer, glioma, glioblastoma, patient survival score, blood serum

## Abstract

Gliomas account for approximately 70% of primary brain tumors in adults. Of all gliomas, grade IV astrocytoma, also called glioblastoma, has the poorest overall survival, with <5% of patients surviving five years after diagnosis. Due to the aggressiveness, lethal nature, and impaired surgical accessibility of the tumor, early diagnosis of the tumor and, in addition, prediction of the patient’s survival time are important. We hypothesize that combining the protein level values of highly recognizable glioblastoma serum biomarkers could help to achieve higher specificity and sensitivity in predicting glioma patient outcome as compared to single markers. The aim of this study was to select the most promising astrocytoma patient overall survival prediction variables from five secretory proteins—glial fibrillary acidic protein (GFAP), matrix metalloproteinase-2 (MMP-2), chitinase 3-like 1 (CHI3L1), osteopontin (OPN), and amphiregulin (AREG)—combining them with routinely used tumor markers to create a Patient Survival Score calculation tool. The study group consisted of 70 astrocytoma patients and 31 healthy controls. We demonstrated that integrating serum CHI3L1 and OPN protein level values and tumor isocitrate dehydrogenase 1 *IDH1* mutational status into one parameter could predict low-grade astrocytoma patients’ two-year survival with 93.8% accuracy.

## 1. Introduction

Gliomas account for approximately 70% of primary brain tumors in adults [1]. The most common glioma type is astrocytoma, named for the shape-form cells—astrocytes—which they originate from. Even low-grade astrocytoma can progress to a more malignant one. Of all gliomas, grade IV astrocytoma, also called glioblastoma (GBM), has the poorest overall survival, with <5% of patients surviving five years after diagnosis [2]. Due to its aggressiveness and the lack of effective treatment, it is very important to predict the course of the disease and patient overall survival. Although several potential diagnostic and prognostic astrocytoma molecular markers have been found, their demonstrated individual potential is not enough. We hypothesized that combining several serum biomarkers could be a good choice to achieve higher specificity and sensitivity in predicting patient outcome.

The best explored potential GBM molecular marker is the chitinase 3-like 1 (CHI3L1) protein, also known as YKL-40. CHI3L1, a 40-kDa secreted glycoprotein, with its gene located on chromosome 1q32.1, is produced by cancer cells and inflammatory cells and has a role in inflammation, cell proliferation, differentiation, protection against apoptosis, stimulation of angiogenesis, and regulation of extracellular tissue remodeling [3]. One of the first demonstrated correlations between CHI3L1 expression and astrocytoma grade was established in 2002 by Tanwar and co-workers [4] and later by other groups [5,6,7,8].

A second potential marker, amphiregulin (AREG), is known to stimulate the proliferation of many cell types. This effect is mainly mediated through its binding and activation of the epidermal growth factor receptor (EGFR) [9]. AREG can also be expressed by multiple populations of activated immune cells in a variety of inflammatory conditions [10]. It was observed that AREG concentrations were elevated in hepatocellular carcinoma patients’ blood serum compared with a healthy control group [11]. In terms of glioma, using microarray analysis, an 11.2 mean fold change overexpression of the AREG gene in glioblastoma relative to pilocytic astrocytoma has been reported [12]. Also, it was shown that AREG overexpression is related to glioma cell line resistance to cannabinoid therapies, which induce apoptosis of tumor cells [13].

Matrix metalloproteinase-2 (MMP-2) is a 72-kDa, Zn+2-dependent secreted or membrane-bound endopeptidase with multiple potential roles in cell proliferation, migration, and invasion and angiogenesis [14]. Glioma cells constantly secrete MMP-2. It was shown that MMP-2 protein expression in brain tumor (including GBM) patients’ cerebrospinal fluid and urine is elevated compared to that of healthy controls [15].

Glial fibrillary acidic protein (GFAP) is also established in the GBM research field. The main function of GFAP is structural as it is an intermediate filament protein that is primarily expressed in astrocytes [16]. It was demonstrated that GFAP levels were significantly higher in glioma patient blood serum when compared with the serum GFAP level in non-glial-origin tumors [17]. Despite the huge number of studies on GFAP’s potential as a GBM prognostic and diagnostic marker, more scientists have discussed GFAP’s application in clinics due to the variety of its isoforms and functional and morphological differences among GFAP-positive cell populations [18].

Osteopontin (OPN) is a highly phosphorylated and glycosylated protein containing the arginyl–glycyl–aspartic acid (RGD) motif. This protein has diverse functions, including functions in bone remodeling, inflammation, cell migration, adhesion, and survival in many cell types [19,20]. A high OPN concentration in GBM patient blood serum is related with poor prognosis [21].

All five investigated proteins were selected based on a detailed literature review [7,21,22,23,24,25] and our earlier laboratory findings [26,27]. However, other variables reported to be associated with patient overall survival and routinely measured, like tumor malignancy, O6-methylguanine-DNA methyltransferase (*MGMT*) promoter methylation status [28,29], isocitrate dehydrogenase 1 (*IDH1*) mutational status [30], and patient gender and age, were also included when calculating patients’ survival probability with the purpose to significantly improve it. Based on previously published studies utilizing glioma patients’ urine, serum, and cerebrospinal fluid circulating marker analysis [22], we believe that combining GFAP, MMP-2, CHI3L1, OPN, and AREG expression values in patient serum will allow us to achieve this study aim: to select the most promising astrocytoma patient overall survival prediction variables from five secretory proteins—GFAP, MMP-2, CHI3L1, OPN, and AREG—and combine them with tumor markers to create a Patient Survival Score (PSS) calculation tool.

## 2. Materials and Methods

This study was approved by the Kaunas Regional Biomedical Research Ethics Committee (No. P2-9/2003) and conforms to The Code of Ethics of the World Medical Association (Declaration of Helsinki).

### 2.1. Study Group and Blood Collection

Patient and healthy control serum samples were collected between 2015 and 2017 with written informed consent from all participants in the Neurosurgery Department of the Hospital of the Lithuanian University of Health Sciences in Kaunas (Lithuania). Patient diagnosis was confirmed according to the 2007 World Health Organization classification [31] and later classified/reclassified according to the 2016 World Health Organization classification [32]. The study group consisted of 70 astrocytoma patients, including 15 *IDH1*-mutant WHO grade II diffuse astrocytoma, 5 *IDH1*-wildtype WHO grade II diffuse astrocytoma, 3 *IDH1*-mutant WHO grade III anaplastic astrocytoma, 1 *IDH1-*wildtype WHO grade III anaplastic astrocytoma, and 46 GBM (35 glioblastoma *IDH1*-wildtype, 5 glioblastoma *IDH*-mutant, and 6 GBM NOS (not otherwise specified)). In further analysis, all grade II astrocytoma cases were assigned to the low-malignancy astrocytoma group (LGG), and grade III cases, together with GBM, were assigned to the high-malignancy astrocytoma group (HGG). Blood from patients was collected before resection of the tumor. For the healthy control group, 31 individuals were chosen with no indications of astrocytoma or any other cancer type, as well as no symptoms of inflammation or infection the day blood was collected. For both groups, a 3 mL vacutainer system of blood collection was used. Within an hour, the tube with blood was centrifuged at 1300× *g* for 10 min, and the supernatant was stored at −80 °C.

### 2.2. Protein Concentration Measurement

The protein expression in blood serum was analyzed using commercial ELISA kits: for AREG protein expression, the Ray BioTech kit (Cat. No ELH-AR) was used; for OPN, the Abcam ab192143 kit; for MMP-2, the Thermo Fisher Scientific kit KHC3081; and for CHI3L1 and GFAP, R&DSystems kits DY2599 and DY2594-05, respectively. For each protein analysis, different serum dilutions were used according to the manufacturers’ recommendations and/or earlier performed analyses [4,5,17,21,33,34]. For CHI3L1 protein analysis, serum samples were diluted 50-fold; for AREG, 2-fold; for GFAP, 3-fold; for MMP-2, 15-fold; and for OPN, 100-fold. Diluted samples were measured in duplicate. Due to unequal serum quantity extractions from the same blood amount and excessively high standard deviations in some samples during experiments, in some patient samples, not all five protein expression levels of interest were established. All other experiment steps and data analysis were performed according to the manufacturers’ recommendations [35,36,37,38,39]. Optical densities at a wavelength of 450 nm were measured using a “Sunrise” absorbance reader (Tecan Trading).

### 2.3. Statistical Analysis

For statistical analysis, software systems IBM SPSS Statistics 22 (IBM SPSS) and GraphPad “Prism 6” were used. Data normality was checked using the Kolmogorov–Smirnov test. Depending on the test results, for normally distributed data, parametric Student’s *t*-test was used to evaluate the differences in protein level in patients’ blood serum in two independent groups. For analysis with at least one comparison with a non-normal distribution of data, the non-parametric Mann–Whitney test was applied. The Kaplan–Meier method was used to evaluate patient survival starting from the date of operation until death, or the date of project closure. Data for survival analysis were divided into two groups according to the median values. To examine the proteins’ prognostic potential, Cox regression analysis was used. First, the five protein serum expression values were tested individually using Cox univariate analysis (the Enter method). Significant variables were included in multivariate Cox analysis (the Backward Conditional method), and the hazard ratios of the remaining targets of the last step were used to calculate the “Patient Survival Score” (PSS) following Zhou [40] with some modifications:

$$\sum_{i=1}^{n}\pm^{*}\frac{HR{\left(target1\right)}^{\pm 1**}xtarget1}{Average}$$

where *HR*(*target*1) is the hazard ratio of target 1 from multivariate Cox regression analysis; *x target*1 is the value of the estimated biomarker, which was recognized as significant in Cox analysis; * + (plus) was used for variables with a positive impact on patient survival (*HR* < 1), and the inverse; **—(minus) was used for variables with positive impact on patient survival (*HR* < 1), and the inverse.

The Patient Survival Score (PSS) according to the same methodology was also calculated including variables like tumor malignancy, *MGMT* promoter methylation status, *IDH1* mutation status, gender, and age.

To estimate the Patient Survival Score sensitivity and specificity for two-year survival prediction, ROC (receiver operating characteristic) curve analysis was used. A significance level of 0.05 was selected to test the statistical hypothesis. The accuracy of the test as a percentage was calculated by dividing the number of correctly assigned values by the total number of values and multiplying by 100.

## 3. Results

### 3.1. Protein Serum Concentration Dependence on Tumor Grade and IDH Mutational Status within Grades

**Figure 1:**
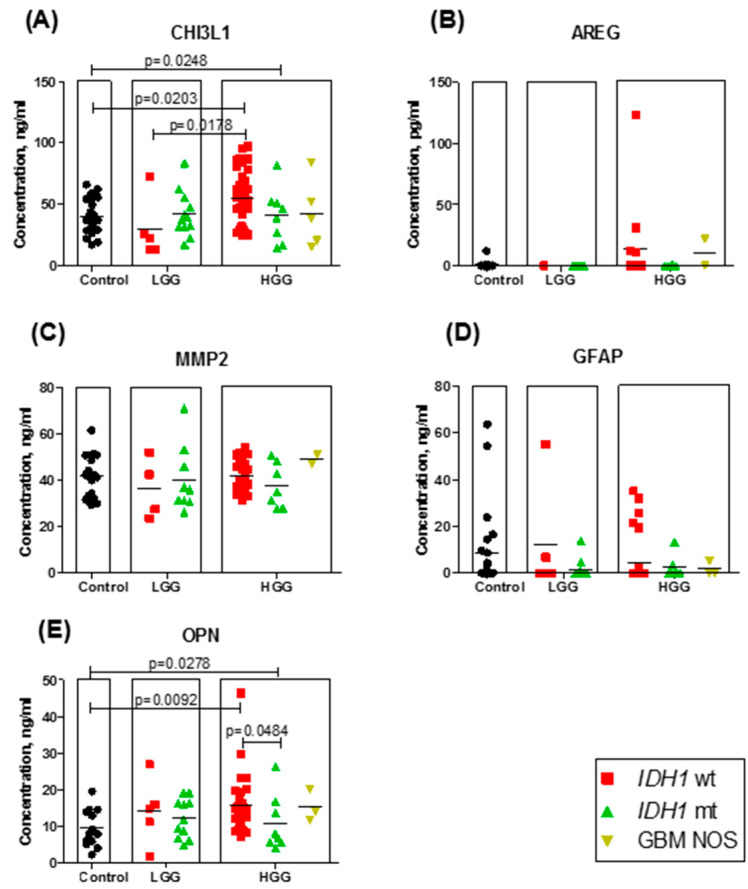
Protein expression in astrocytoma patient and healthy control blood serum; *IDH1* mutational status is highlighted with different colors (red—*IDH1*-wildtype, green—*IDH1*-mutant, yellow—GBM NOS): (**A**) chitinase 3-like 1 (CHI3L1) (*n* = 99); (**B**) amphiregulin (AREG) (*n* = 38); (**C**) matrix metalloproteinase-2 (MMP-2) (*n* = 61); (**D**) glial fibrillary acidic protein (GFAP) (*n* = 83); (**E**) osteopontin (OPN) (*n* = 69). The lines in graphs correspond to the medians.

As mentioned above, in our study, five potential astrocytoma/GBM molecular markers were chosen, and their expression in astrocytoma patients’ blood sera was investigated using ELISA methods. First, the CHI3L1 protein level in different patients’ sera was evaluated. The median expression level of the protein was higher in HGG compared with the control group (*p* = 0.0248) (Figure 1A). The average expression levels in the control group, LGG, and HGG (III and IV grade) were 40.4 ng/mL (range: 17.2 to 66.1), 41.3 ng/mL (range: 13.2 to 107.0), and 51.1 ng/mL (range: 14.0 to 96.9). Dividing the LGG and HGG groups by their *IDH1* mutational status, statistically significant differences were observed between the control and HGG (astrocytoma grade III and IV) groups as well as the HGG *IDH1*-wildtype and LGG *IDH1*-wildtype groups (*p* = 0.0203 and *p* = 0.0178 respectively) (Figure 1A). Furthermore, approximately one-quarter (27.7%) of all high-grade *IDH1*-wildtype astrocytoma patients had higher CHI3L1 protein concentrations in serum than the highest CHI3L1 value in the control group (66.1 ng/mL).

Next, the AREG concentrations in different *IDH1* mutational status and different malignancy astrocytoma group samples and healthy control group serum samples are presented in Figure 1B. There were no statistically significant differences observed between any groups. The MMP-2 (Figure 1C) and GFAP (Figure 1D) proteins did not demonstrate any statistically significant expression differences between different malignancy grades and different *IDH1* mutational status astrocytoma groups, or between the astrocytoma and healthy control groups.

The concentration values of OPN are shown in Figure 1E. OPN demonstrated a statistically significant protein expression difference in blood serum between the control and HGG groups. The averaged expression differed from 9.6 ng/mL (range: 2.3 to 19.6) in the control group to 14.6 ng/mL (range: 4.0 to 46.4) in the HGG group. No significant difference was noted between the LGG and control groups, or between LGG and HGG. Like in the CHI3L1 case, dividing the OPN LGG and HGG groups by their *IDH1* mutational status, OPN demonstrated a statistically significant protein expression increase in HGG *IDH1*-wildtype as compared to the control group (*p* = 0.0092). The median expression differed from 8.0 ng/mL (range: 2.3 to 19.6) in the control group to 13.8 ng/mL (range: 7.3 to 46.4) in the high-grade *IDH1*-wildtype astrocytoma group. Interestingly, in HGG, a statistically significant difference was also observed between the *IDH1*-wildtype and *IDH1*-mutant groups (*p* = 0.0484), with a lower concentration in *IDH1*-mutant. No significant difference was noted between any other groups.

### 3.2. Protein Serum Concentrations Associated with Patient Survival

**Figure 2:**
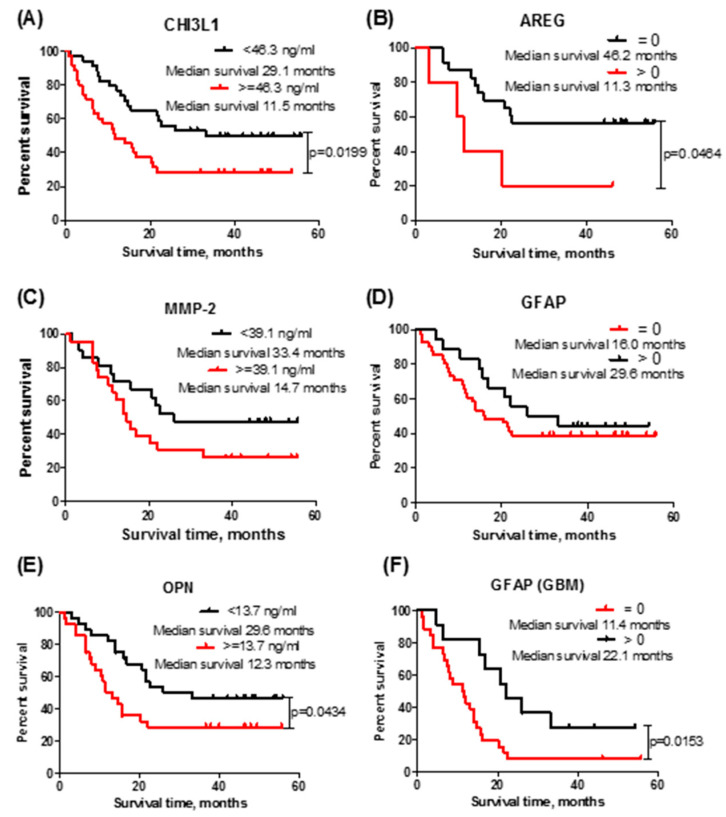
Kaplan–Meier survival curves, indicating survival (months) dependency on median protein expression levels in patient serum: (**A**) CHI3L1 (*n* = 69). Log-rank test; χ^2^ = 5.417; *df* = 1; *p* = 0.0199; (**B**) AREG (*n* = 28). Log-rank test; χ^2^ = 3.968; *df* = 1; *p* = 0.0464; (**C**) MMP-2 (*n* = 44). Log-rank test; χ^2^ = 2.264; *df* = 1; *p* = 0.1324; (**D**) GFAP (*n* = 59). Log-rank test; χ^2^ = 0.768; *df* = 1; *p* = 0.3808; (**E**) OPN (*n* = 56). Log-rank test; χ^2^ = 4.080; *df* = 1; *p* = 0.0434; (**F**) GFAP in the glioblastoma group (*n* = 37). Log-rank test; χ^2^ = 5.880; *df* = 1; *p* = 0.0153.

Kaplan–Meier analysis showed that lower CHI3L1 serum concentration is related to longer astrocytoma patient overall survival (Log-rank test; χ^2^ = 5.417; *df* = 1; *p* = 0.0199) (Figure 2A), while higher AREG concentration is related to worse survival prognosis (Log-rank test; χ^2^ = 3.968; *df* = 1; *p* = 0.0464).

Astrocytoma patient survival’s dependence on MMP-2 and GFAP concentrations is shown in Figure 2C,D. No statistically significant associations were estimated (MMP-2: Log-rank test; χ^2^ = 2.264; *df* = 1; *p* = 0.1324; GFAP: Log-rank test; χ^2^ = 0.768; *df* = 1; *p* = 0.3808). When investigating OPN expression’s association with patient survival, a significant relation was found: a lower than median (13.7 ng/mL) OPN concentration was related to longer patient survival (Log-rank test; χ^2^ = 4.080; *df* = 1; *p* = 0.0434) (Figure 2E). However, when investigating only GBM patients’ survival in the low- and high-GFAP-expression groups (Figure 2F), it was observed that lower GFAP concentration was related with shorter GBM patient survival (Log-rank test; χ^2^ = 5.880; *df* = 1; *p* = 0.0153).

### 3.3. The Patient Survival Score in Predicting Glioma Patient Outcome

To evaluate the prognostic potential of the investigated serum proteins individually, Cox regression analysis was used.

Univariate Cox regression analysis identified the three most promising proteins—CHI3L1, AREG, and OPN—as being significantly associated with death risk. Multivariate analysis selected two of them—CHI3L1 and OPN—and the Patient Survival Score 0 (PSS0) was calculated. Astrocytoma patient survival’s dependence on this score was checked. Although patients with low PSS0 lived significantly shorter than did those with higher scores (Log-rank test; χ^2^ = 7.076; *df* = 1; *p* = 0.008), the score showed only little improvement as compared to the individual CHI3L1 or OPN survival curves.

Next, the same Cox regression analysis was repeated including clinical variables, like tumor malignancy, *MGMT* promoter methylation status, *IDH1* mutation status, and patient gender and age (<50 or ≥50 years) (Table 1). The variables selected as significant after univariate analysis were the CHI3L1 and OPN expression levels in patient serum, tumor *IDH1* mutational status, patient age, and malignancy grade. Patient malignancy grade and age were excluded from the analysis in Steps 1 and 2 for their lack of power in predicting patient death risk.

**Figure 3:**
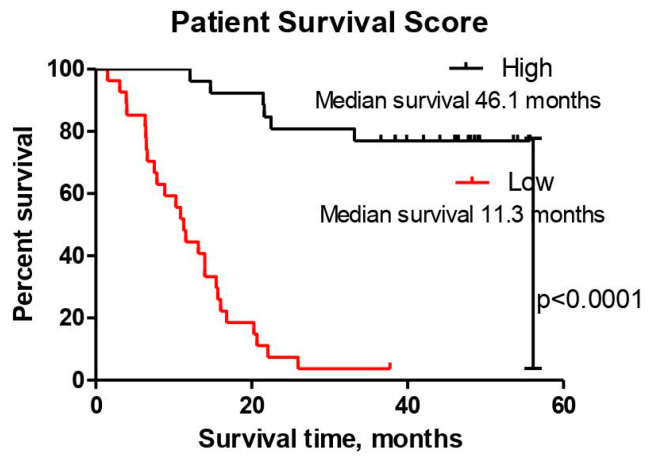
Kaplan–Meier survival dependency on the calculated median Patient Survival Score (*n* = 53). Log-rank test; χ^2^ = 42.630; *df* = 1; *p* < 0.0001.

The individual Patient Survival Score (PSS) was calculated as described in Section 2.3, and Kaplan–Meier analysis was performed. The variables included in the Patient Survival Score calculation were as follows: CHI3L1, OPN, and *IDH1* mutational status. The Patient Survival Score values ranged from −5.30 to 8.19, being higher for LGG patients than for HGG patients, and reached mean values in these groups of 4.34 (range: −2.77 to 8.01) and −0.24 (range: −5.30 to 8.19), respectively. This showed that a higher than median Patient Survival Score is related to longer overall survival of astrocytoma group patients (Log-rank test; χ^2^ = 42.630; *df* = 1; *p* < 0.001) (Figure 3) and is highly significant when combining the three chosen variables, rather than using each one individually (Figure 2A,E; data of individual *IDH1* mutational status Kaplan–Meier analysis are not shown (Log-rank test; χ^2^ = 27.289; *df* = 1; *p* < 0.001)).

**Figure 4:**
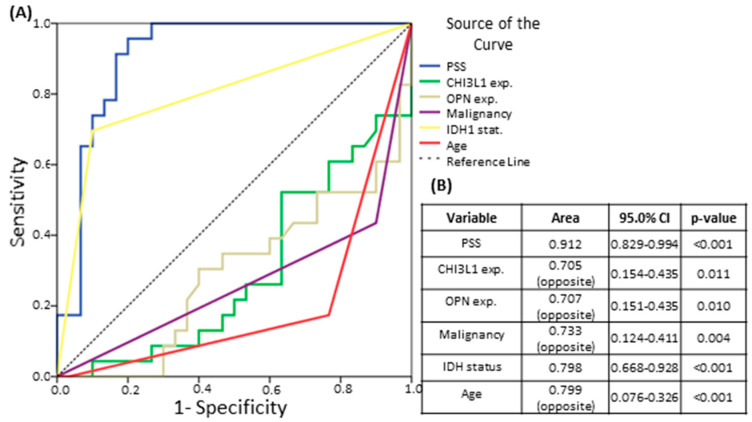
Receiver operating characteristic (ROC) curves of different variables’ ability to predict astrocytoma patients’ 24-month survival: (**A**) ROC curves of the Patient Survival Score (PSS), CHI3L1 and OPN expression levels, tumor malignancy grade, *IDH1* mutational status, and patient age; (**B**) Table with the investigated variables’ ROC curve areas, 95% confidence intervals (CIs), and *p*-values.

ROC curve analysis was applied to evaluate the Patient Survival Score’s sensitivity and specificity on two-year astrocytoma patient survival prediction. It demonstrated significance with an area value of 0.912 (95% CI: 0.829–0.994), which is rated as a “perfect test”. The sensitivity of the test reached 95.7% and its specificity reached 80.0% (Figure 4A), with the Patient Survival Score value of −1.6794 selected as a break point. The same analysis was performed for the GBM and grade II astrocytoma groups. The patient survival score sensitivity was 100% in both groups, with specificity of 74.1% in the GBM group and 66.7% in the grade II astrocytoma group. The area values for the test were 0.894 (95% CI: 0.786–1.000) and 0.718 (95% CI: 0.259–1.000), respectively.

ROC curve analysis was also performed to evaluate all other variables that demonstrated significance in Univariate Cox regression analysis—individual CHI3L1 and OPN expressions, tumor malignancy grade, patient age, and *IDH1* mutational status—in terms of their reliability in predicting the 24-month survival of the patient (Figure 4). As expected, the PSS demonstrated the best prediction potential compared with all other investigated variables.

### 3.4. The Patient Survival Score’s Ability to Predict Survival in Comparison to IDH Mutational Status

It is well known that *IDH* mutational status is associated with patient overall survival [30]; due to this fact, to evaluate the Patient Survival Score’s true potential to predict astrocytoma patients’ survival, it is essential to test how *IDH1* mutational status alone can predict it. The PSS’s accuracy in predicting astrocytoma patients’ two-year survival was found to be 86.8%, while the accuracy of prediction using *IDH1* mutational status was 81.1%. Dividing patients into groups depending on their astrocytoma malignancy grade, no improvement was observed in the GBM group (83.8% in both groups); however, in the grade II astrocytoma group, PSS demonstrated a solid improvement in predicting two-year survival compared to *IDH1* mutational status alone—93.8% vs. 75.00%, respectively.

## 4. Discussion

The vast majority of scientific research and applications of new therapies for astrocytoma are intended to cure patients or prolong their survival, but despite all efforts of scientists and clinicians, this diagnosis remains one of the most devastating and mostly lethal. A more accurate prediction of survival time could help us to better understand the course of astrocytoma and accept it. Here we investigated five potential astrocytoma molecular markers’ expression levels in patient blood serum to establish their potential for diagnosing astrocytoma and predicting patient overall survival. We created an astrocytoma patient survival prediction model consisting of CHI3L1 and OPN expression levels and patient *IDH1* mutational status, which predicted two-year astrocytoma patient survival with accuracy rates of 86.8% in all astrocytomas and 93.8% in low-grade astrocytomas (LGG).

The first protein, CHI3L1, in our study was chosen because many earlier studies showed this protein’s potential to distinguish GBM from healthy controls [41]. Our results reinforce these studies: the measured average CHI3L1 concentration in the control group was lower compared to that for high-grade astrocytoma (40.4 ng/mL in low- and 51.1 ng/mL in high-grade astrocytoma (III and IV grade)). Other authors published mean CHI3L1 concentrations ranging from 25.6 ng/mL to 84.5 ng/mL in healthy control groups and from 75.8 ng.ml to 159.2 ng/mL in GBM patients’ blood serum or plasma samples [4,5,6,7,8]. Despite little discrepancy in CHI3L1′s ability to distinguish GBM patient groups from healthy controls, this protein demonstrated a reliable (*p* = 0.02) difference (Figure 2A) in patient overall survival based on the median CHI3L1 value. These results confirm Barnardi and co-workers’ findings [7]. Akiyama and his group (2014) showed that the CHI3L1 level in the supernatant of the Temozolomide-resistant U87 cell line was upregulated several-fold compared with the level of the parental U87 cell line, and CHI3L1 gene inhibition partially restored sensitivity to Temozolomide [42], which alerts us to the possibility that CHI3L1 might be useful not only as a potential astrocytoma marker but also as a promising therapeutic candidate.

For this investigation, the AREG protein was chosen due to the earlier laboratory findings of Steponaitis and colleagues’ analysis. It was demonstrated that AREG mRNA expression in astrocytoma tumor tissue was undetectable in more than 1/3 of the samples (the signal was under the sensitivity threshold), and in those above the sensitivity threshold, levels were elevated in the GBM group [27]. Ishikawa and colleagues examined AREG concentrations in the blood serum of non-small cell lung cancer patients treated with Gefitinib—an orally administered inhibitor of EGFR tyrosine kinase—and like in our study, they demonstrated a dependency between higher AREG concentrations and shorter patient overall survival, which is probably related with the EGFR signaling pathway involved in the proliferation, invasion, and survival of cancer cells [43].

Another protein investigated in this study was MMP-2. Some groups investigated MMP-2 protein expression in astrocytic brain tissue. MMP-2 expression was found to be significantly higher in GBM compared to normal brain tissue (*p* < 0.001), diffuse astrocytoma (*p* < 0.001), and anaplastic astrocytoma (*p* < 0.05). Also, in the same study, Ramachandran and colleagues found MMP-2 expression to be associated with shorter overall survival in patients with grade II–IV astrocytic tumors [44]. On the other hand, our study did not demonstrate any statistically significant differences in protein expression in serum between astrocytoma grades and the control group, and no relationship of elevated MMP-2 protein expression with patient survival time.

GFAP is better characterized as a potential astrocytoma biomarker than the previously discussed proteins. Since the first published article of GFAP’s associations with astrocytoma in 1972 [45], there have been dozens of studies about how this protein interfaces with astrocytoma. Contrary to our results, many groups showed elevated GFAP levels in GBM patients’ sera compared with healthy controls and sera from patients with other brain pathologies. Jung and colleagues demonstrated detectable GFAP values in 40 of 50 GBM patients’ serum samples (median: 0.18 μg/L; range: 0 to 5.6 μg/L) using ELISA test, when non-GBM tumor patients and all healthy subjects showed zero serum GFAP levels in all samples [17]. Other groups also demonstrated significantly higher GFAP values in GBM patients compared to all other tumors, or lower-malignancy-grade astrocytoma [46,47]. On the other hand, Lange’s group did not show significantly higher levels in grade IV astrocytoma patient plasma [48]. The same group’s results also reiterate our findings that detectable GFAP serum levels show no dependencies with longer astrocytoma patient survival time [48]. All these discrepancies between different groups’ results could be explained by the huge variety of GFAP isoforms, post-translational modifications [49], and functional and morphological differences of GFAP-positive cell populations [18], and all these factors must be better characterized in further research investigating GFAP’s potential as an astrocytoma diagnostic marker.

The last examined protein, OPN, is also better investigated as a potential astrocytoma marker than AREG and MMP-2. In Zhao and colleagues’ meta-analysis, they demonstrated that in patients with high-grade glioma tumor, the OPN gene and protein expression was significantly higher than that in patients with low-grade glioma [50]. Their analysis also demonstrated a correlation between high OPN expression and tumor reoccurrence and indicated that OPN expression was significantly related to overall survival [50]. In Sreekanthreddy’s study, elevated serum OPN levels were shown: in GBM, it was 31.54 ± 28.98 ng/mL, compared with 17.38 ± 7.91 ng/mL in grade III astrocytoma, 13.79 ± 4.56 ng/mL in grade II astrocytoma, and 11.70 ± 7.26 ng/mL in normal controls. The same group also showed that GBM patients with high serum OPN levels had poorer survival than did those with low serum OPN levels [21]. Our results reiterate these findings, and all together they could possibly be explained by the claim of Denhardt and colleagues that tumor cells produce OPN that could protect the tumor cells themselves against attack by macrophages by suppressing their production of nitric oxide (NO), which can kill tumor cells [51].

Since proteomics approaches have been applied in science, many protein-based assays have been implemented in clinics for early diagnosis, monitoring of treatment, and subtyping for many different diseases, including cancer [52]. However, despite several promising discoveries, there is no efficient individual serum/plasma molecular marker application for astrocytoma diagnostics and prognostics in a clinical setting. In 2011, Elstner and colleagues published an article [53] in which none of 14 investigated serum proteins were sufficiently specific and sensitive to serve as a potential GBM molecular marker. However, their serum protein profile formed of more than one protein—bone morphogenic protein 2 (BMP2), heat shock 70-kDa protein (HSP70), and chemokine (CXC motive) ligand 10 (CXCL10)—enabled correct assignment to the GBM group from the control group with specificity and sensitivity of 89% and 96%, respectively. Other groups also demonstrated the impact of combined protein expression in glioma prognostics and diagnostics: a profile consisting of thrombospondin-1 (TSP1), HSP70, and insulin-like growth factor binding protein-3 (IGFBP3) was able to correctly predict 15-month survival after tumor resection in all cases using a decision tree method [53]; a panel composed of four cytokines—interleukin 7 (IL-7), interleukin-1 receptor-like 1 (IL1R4/ST2), soluble glycoprotein 130 (sgp130), and monocyte chemoattractant protein-1 (MCP-1)—showed a significant correlation with overall survival in multivariate Cox regression analysis [54]; analysis by Perez-Larraya’s group discriminated GBM and nonglial brain tumors [55]. Nijaguna and colleagues used an 18-cytokine signature and demonstrated high efficiency in distinguishing GBM, grade III, and grade II astrocytoma serum samples from those of healthy individuals [56]. Our group demonstrated that integrating the values of CHI3L1 and OPN protein expression in serum with *IDH1* mutational status into one parameter could predict patient 24-month survival with 86.8% accuracy for astrocytoma patients of all grades. However, only in grade II astrocytoma when comparing the PSS’s predicting accuracy with that of individual parameters of PSS (*IDH1* mutational status) was a significant improvement demonstrated and 93.8% accuracy reached.

## 5. Conclusions

These results lead to the conclusion that combining several disease-specific tumor and serum biomarkers could be a good choice to achieve higher specificity and sensitivity in predicting low-grade astrocytoma patient outcome.

## Figures and Tables

**Table 1:** Univariate and multivariate Cox regression analysis.

Univariate Cox Regression				Multivariate Cox Regression			
Factor	HR	95.0% CI for HR	*p*-Value	Step	HR	95.0% CI for HR	*p*-Value
Age	6.866	3.436–13.729	<0.001				
Gender	1.310	0.726–2.361	0.370				
Malignancy	7.854	2.804–21.998	<0.001				
*MGMT* methylation	0.793	0.429–1.466	0.459				
*IDH1* status	6.786	2.828–16.287	<0.001	3	0.113	0.038–0.339	<0.001
CHI3L1 expression	1.013	1.001–1.025	0.039	3	1.023	1.007–1.040	0.005
OPN expression	1.094	1.046–1.144	<0.001	3	1.092	1.038–1.149	0.001
AREG expression	1.050	1.009–1.093	0.017				
MMP-2 expression	1.013	0.983–1.051	0.343				
GFAP expression	1.005	0.974–1.038	0.742				

## References

[1] Hofer S., Rushing E., Preusser M., Marosi C. (2014). Molecular biology of high-grade gliomas: What should the clinician know?. Chin. J. Cancer.

[2] Ostrom Q.T., Bauchet L., Davis F.G., Deltour I., Fisher J.L., Langer C.E., Pekmezci M., Schwartzbaum J.A., Turner M.C., Walsh K.M. (2014). The epidemiology of glioma in adults: A “state of the science” review. Neuro. Oncol..

[3] Johansen J.S., Schultz N.A., Jensen B. (2009). V Plasma YKL-40: A potential new cancer biomarker?. Future Oncol..

[4] Tanwar M.K., Gilbert M.R., Holland E.C. (2002). Gene expression microarray analysis reveals YKL-40 to be a potential serum marker for malignant character in human glioma. Cancer Res..

[5] Hormigo A., Gu B., Karimi S., Riedel E., Panageas K.S., Edgar M.A., Tanwar M.K., Rao J.S., Fleisher M., DeAngelis L.M. (2006). YKL-40 and matrix metalloproteinase-9 as potential serum biomarkers for patients with high-grade gliomas. Clin. Cancer Res..

[6] Kazakova M.H., Staneva D.N., Koev I.G., Staikov D.G., Mateva N., Timonov P.T., Miloshev G.A., Sarafian V.S. (2014). Protein and mRNA levels of YKL-40 in high-grade glioma. Folia Biol..

[7] Bernardi D., Padoan A., Ballin A., Sartori M.T., Manara R., Scienza R., Plebani M., Della Puppa A. (2012). Serum YKL-40 following resection for cerebral glioblastoma. J. Neurooncol..

[8] Koev I.G., Feodorova Y.N., Kazakova M.H., Staykov D.G., Kitov B.D., Sarafian V.S. (2014). Glioblastoma Multiforme Classified as Mesenchymal Subtype. Folia Med..

[9] Berasain C., Avila M.A. (2014). Amphiregulin. Semin. Cell Dev. Biol..

[10] Zaiss D.M.W., Gause W.C., Osborne L.C., Artis D. (2015). Emerging functions of amphiregulin in orchestrating immunity, inflammation, and tissue repair. Immunity.

[11] Han S.X., Bai E., Jin G.H., He C.C., Guo X.J., Wang L.J., Li M., Ying X., Zhu Q. (2014). Expression and clinical significance of YAP, TAZ, and AREG in hepatocellular carcinoma. J. Immunol. Res..

[12] Marie S.K.N., Okamoto O.K., Uno M., Hasegawa A.P.G., Oba-Shinjo S.M., Cohen T., Camargo A.A., Kosoy A., Carlotti C.G., Toledo S. (2008). Maternal embryonic leucine zipper kinase transcript abundance correlates with malignancy grade in human astrocytomas. Int. J. Cancer.

[13] Lorente M., Carracedo A., Torres S., Natali F., Agia A., Hernanández-Tiedra S., Salazar M., Blázquez C., Guzmán M., Velasco G. (2009). Amphiregulin is a factor for resistance of glioma cells to cannabinoid-induced apoptosis. Glia.

[14] Chang C., Werb Z. (2001). The many faces of metalloproteases: Cell growth, invasion, angiogenesis and metastasis. Trends Cell Biol..

[15] Smith E.R., Zurakowski D., Saad A., Scott R.M., Moses M.A. (2008). Urinary biomarkers predict brain tumor presence and response to therapy. Clin. Cancer Res..

[16] Brenner M. (2014). Role of GFAP in CNS injuries. Neurosci. Lett..

[17] Jung C.S., Foerch C., Schänzer A., Heck A., Plate K.H., Seifert V., Steinmetz H., Raabe A., Sitzer M. (2007). Serum GFAP is a diagnostic marker for glioblastoma multiforme. Brain.

[18] van Bodegraven E.J., van Asperen J.V., Robe P.A.J., Hol E.M. (2019). Importance of GFAP isoform-specific analyses in astrocytoma. Glia.

[19] Lund S.A., Giachelli C.M., Scatena M. (2009). The role of osteopontin in inflammatory processes. J. Cell Commun. Signal..

[20] Sase S.P., Ganu J.V., Nagane N. (2012). Osteopontin: A Novel Protein Molecule. Ind. Med. Gaz..

[21] Sreekanthreddy P., Srinivasan H., Kumar D.M., Nijaguna M.B., Sridevi S., Vrinda M., Arivazhagan A., Balasubramaniam A., Hegde A.S., Chandramouli B.A. (2010). Identification of potential serum biomarkers of glioblastoma: Serum osteopontin levels correlate with poor prognosis. Cancer Epidemiol. Biomark. Prev..

[22] Best M.G., Sol N., Zijl S., Reijneveld J.C., Wesseling P., Wurdinger T. (2015). Liquid biopsies in patients with diffuse glioma. Acta Neuropathol..

[23] Tabouret E., Bertucci F., Pierga J.-Y., Petit T., Levy C., Ferrero J.-M., Campone M., Gligorov J., Lerebours F., Roché H. (2016). MMP2 and MMP9 serum levels are associated with favorable outcome in patients with inflammatory breast cancer treated with bevacizumab-based neoadjuvant chemotherapy in the BEVERLY-2 study. Oncotarget.

[24] Reiss Y., Machein M.R., Plate K.H. (2005). The role of angiopoietins during angiogenesis in gliomas. Brain Pathol..

[25] Güttler A., Giebler M., Cuno P., Wichmann H., Keßler J., Ostheimer C., Söling A., Strauss C., Illert J., Kappler M. (2013). Osteopontin and splice variant expression level in human malignant glioma: Radiobiologic effects and prognosis after radiotherapy. Radiother. Oncol..

[26] Sincevičiūtė R., Vaitkienė P., Urbanavičiūtė R., Steponaitis G., Tamašauskas A., Skiriutė D. (2018). MMP2 is associated with glioma malignancy and patient outcome. Int. J. Clin. Exp. Pathol..

[27] Steponaitis G., Kazlauskas A., Skiriute D., Vaitkiene P., Skauminas K., Tamasauskas A. (2019). Significance of Amphiregulin (AREG) for the Outcome of Low and High Grade Astrocytoma Patients. J. Cancer.

[28] Håvik A.B., Brandal P., Honne H., Dahlback H.S.S., Scheie D., Hektoen M., Meling T.R., Helseth E., Heim S., Lothe R.A. (2012). MGMT promoter methylation in gliomas-assessment by pyrosequencing and quantitative methylation-specific PCR. J. Transl. Med..

[29] Fiano V., Trevisan M., Trevisan E., Senetta R., Castiglione A., Sacerdote C., Gillio-Tos A., De Marco L., Grasso C., Magistrello M. (2014). MGMT promoter methylation in plasma of glioma patients receiving temozolomide. J. Neurooncol..

[30] Lv S., Teugels E., Sadones J., Quartier E., Huylebrouck M., Du Four S., Le Mercier M., De Witte O., Salmon I., Michotte A. (2011). Correlation between IDH1 gene mutation status and survival of patients treated for recurrent glioma. Anticancer Res..

[31] Louis D.N., Ohgaki H., Wiestler O.D., Cavenee W.K., Burger P.C., Jouvet A., Scheithauer B.W., Kleihues P. (2007). The 2007 WHO classification of tumours of the central nervous system. Acta Neuropathol..

[32] Louis D.N., Perry A., Reifenberger G., von Deimling A., Figarella-Branger D., Cavenee W.K., Ohgaki H., Wiestler O.D., Kleihues P., Ellison D.W. (2016). The 2016 World Health Organization Classification of Tumors of the Central Nervous System: A summary. Acta Neuropathol..

[33] Peterson E.A., Shabbeer S., Kenny P.A. (2012). Normal range of serum Amphiregulin in healthy adult human females. Clin. Biochem..

[34] Kai H., Ikeda H., Yasukawa H., Kai M., Seki Y., Kuwahara F., Ueno T., Sugi K., Imaizumi T. (1998). Peripheral blood levels of matrix metalloproteases-2 and -9 are elevated in patients with acute coronary syndromes. J. Am. Coll. Cardiol..

[35] RayBiotech Human Amphiregulin ELISA. https://www.raybiotech.com/human-ar-amphiregulin-elisa-kit-available-serum-plasma-cell-culture-supernatant-and-urine/.

[36] Abcam Human Osteopontin ELISA Kit (ab192143). https://www.abcam.com/human-osteopontin-elisa-kit-ab192143.html.

[37] MMP2 Human ELISA Kit. https://www.thermofisher.com/elisa/product/MMP2-Human-ELISA-Kit/KHC3081.

[38] Human GFAP DuoSet ELISA. https://www.rndsystems.com/products/human-gfap-duoset-elisa_dy2594-05.

[39] Human Chitinase 3-like 1 DuoSet ELISA. https://www.rndsystems.com/products/human-chitinase-3-like-1-duoset-elisa_dy2599.

[40] Zhou M., Zhang Z., Zhao H., Bao S., Sun J. (2018). A novel lncRNA-focus expression signature for survival prediction in endometrial carcinoma. BMC Cancer.

[41] Zhao Y.-H., Pan Z.-Y., Wang Z.-F., Ma C., Weng H., Li Z.-Q. (2018). YKL-40 in high-grade glioma: Prognostic value of protein versus mRNA expression. Glioma.

[42] Akiyama Y., Ashizawa T., Komiyama M., Miyata H., Oshita C., Omiya M., Iizuka A., Kume A., Sugino T., Hayashi N. (2014). YKL-40 downregulation is a key factor to overcome temozolomide resistance in a glioblastoma cell line. Oncol. Rep..

[43] Ishikawa N., Daigo Y., Takano A., Taniwaki M., Kato T., Hayama S., Murakami H., Takeshima Y., Inai K., Nishimura H. (2005). Increases of amphiregulin and transforming growth factor-α in serum as predictors of poor response to gefitinib among patients with advanced non-small cell lung cancers. Cancer Res..

[44] Ramachandran R.K., Sørensen M.D., Aaberg-Jessen C., Hermansen S.K., Kristensen B.W. (2017). Expression and prognostic impact of matrix metalloproteinase-2 (MMP-2) in astrocytomas. PLoS ONE.

[45] Uyeda C.T., Eng L.F., Bignami A. (1972). Immunological study of the glial fibrillary acidic protein. Brain Res..

[46] Tichy J., Spechtmeyer S., Mittelbronn M., Hattingen E., Rieger J., Senft C., Foerch C. (2016). Prospective evaluation of serum glial fibrillary acidic protein (GFAP) as a diagnostic marker for glioblastoma. J. Neurooncol..

[47] Ilhan-Mutlu A., Wagner L., Widhalm G., Wöhrer A., Bartsch S., Czech T., Heinzl H., Leutmezer F., Prayer D., Marosi C. (2013). Exploratory investigation of eight circulating plasma markers in brain tumor patients. Neurosurg. Rev..

[48] Lange R.P., Everett A., Dulloor P., Korley F.K., Bettegowda C., Blair C., Grossman S.A., Holdhoff M. (2014). Evaluation of eight plasma proteins as candidate blood-based biomarkers for malignant gliomas. Cancer Investig..

[49] Middeldorp J., Hol E.M. (2011). GFAP in health and disease. Prog. Neurobiol..

[50] Zhao M., Xu H., Liang F., He J., Zhang J. (2014). Association of osteopontin expression with the prognosis of glioma patient: A meta-analysis. Tumor Biol..

[51] Denhardt D.T., Chambers A.F. (1994). Overcoming obstacles to metastasis—Defenses against host defenses: Osteopontin (OPN) as a shield against attack by cytotoxic host cells. J. Cell. Biochem..

[52] Wulfkuhle J.D., Liotta L.A., Petricoin E.F. (2003). Proteomic applications for the early detection of cancer. Nat. Rev. Cancer.

[53] Elstner A., Stockhammer F., Nguyen-Dobinsky T.N., Nguyen Q.L., Pilgermann I., Gill A., Guhr A., Zhang T., Von Eckardstein K., Picht T. (2011). Identification of diagnostic serum protein profiles of glioblastoma patients. J. Neurooncol..

[54] Lin Y., Zhang G., Zhang J., Gao G., Li M., Chen Y., Wang J., Li G., Song S.W., Qiu X. (2013). A panel of four cytokines predicts the prognosis of patients with malignant gliomas. J. Neurooncol..

[55] Pérez-Larraya J.G., Paris S., Idbaih A., Dehais C., Laigle-Donadey F., Navarro S., Capelle L., Mokhtari K., Marie Y., Sanson M. (2014). Diagnostic and prognostic value of preoperative combined GFAP, IGFBP-2, and YKL-40 plasma levels in patients with glioblastoma. Cancer.

[56] Nijaguna M.B., Patil V., Hegde A.S., Chandramouli B.A., Arivazhagan A., Santosh V., Somasundaram K. (2015). An eighteen serum cytokine signature for discriminating Glioma from normal healthy individuals. PLoS ONE.

